# Discovering Protein Receptors for Signaling Nucleotides

**DOI:** 10.1371/journal.ppat.1005569

**Published:** 2016-08-25

**Authors:** Vincent T. Lee

**Affiliations:** Department of Cell Biology and Molecular Genetics, Maryland Pathogen Research Institute, University of Maryland at College Park, College Park, Maryland, United States of America; The Fox Chase Cancer Center, UNITED STATES

Basic research is a journey taken by scientists to explore our fundamental understanding of biology. The process of conducting basic research leads to new solutions and knowledge that can be applied to different problems, including many unexpected ones. One of the primary questions I have been interested in since starting my journey, upon entering graduate school, is: how do bacteria detect changes and modify themselves accordingly to survive diverse environments? This is critical during infection of the host, a situation in which the bacteria detect cues upon entering the host and additional changes during the progression of the infection. Understanding the ways that bacteria sense these changes and respond may reveal potential new strategies to combat infections and antibiotic resistance.

From the beginning of my graduate training in Olaf Schneewind’s lab during the mid-1990s, I knew that there was a growing body of knowledge about signal transduction systems used by bacteria to respond to environmental changes. One class of signal transduction systems, called the second messenger signaling system, detects changes in the environment and generates a second messenger signaling molecule in the cell. This signaling molecule then binds directly to proteins or RNA to alter their behavior, thus allowing the bacterial cell to respond to changes. This fundamental signaling process that occurs in bacteria also exists in higher organisms, so findings from prokaryotes have the potential to explain how signaling systems work in eukaryotes. The literature on secondary signaling systems in bacteria in the 1990s was predominantly based on studies of two molecules, cyclic AMP (cAMP) and “magic spot” (ppGpp), in one model bacterial organism (*Escherichia coli*), indicating that other secondary messenger signaling molecules may exist. Since that time, a large number of other signaling molecules (cyclic-di-GMP, cyclic-di-AMP, cyclic-GMP-AMP, and cyclic GMP, among many others) have been identified by numerous labs, supporting the idea of diversity of second messenger signaling systems in prokaryotic organisms. Three basic research questions can be asked for all of these signaling molecules: 1. Which of the cellular protein(s) and RNA(s) bind these signaling molecules? 2. What are the allosteric changes on the protein and RNA upon binding to the signaling molecule? 3. How does a cell coordinate these events to form a coherent response?

Continuing on this path as a post-doctoral fellow in Steve Lory’s lab, I successfully identified a few of these new binding proteins, which launched the start of my own lab on the quest to find other binding proteins in the bacterial cell. One way to find these proteins was to test every single protein encoded in the genome of a bacterium. This is doable since the number of protein-encoding genes is a discrete, finite number (approximately 1,000 to 6,000, depending on the bacterial species). However, I ran into a technological roadblock, since testing even a few proteins took many months to complete. There were no feasible ways to test thousands of proteins for their ability to bind a signaling molecule in a reasonable time frame (i.e., the tenure clock). On a dark day before the winter holidays after years of searching for a solution, I spotted a mixture of protein and the ligand on a dry nitrocellulose paper. This quick and simple process, akin to putting a paper towel on a coffee spill, easily differentiated binding proteins, since they prevented the signaling molecule from moving with the liquid wicking away on the paper. Through both serendipity and paying careful attention in the lab, I realized that this procedure, which we termed Differential Radial Capillary Action of Ligand Assay, or DRaCALA, that took only seconds to complete was a solution to my technological roadblock.

Work by my student Kevin Roelofs adapted DRaCALA to screen thousands of proteins encoded on the bacterial genome. Members of my lab, including Kevin, Mona Orr, and Sarah Helman, as well as other labs have used this assay to identify a number of new protein receptors for cyclic-di-GMP and cyclic-di-AMP. In addition to these discoveries, my students Ori Lieberman and Eric Zhou have used this method to identify small molecule inhibitors that can occupy the binding site and prevent binding by the signaling molecule. Such inhibitory molecules can serve as lead compounds to treat bacterial infections and reduce antibiotic resistance. In addition, Gregory Donaldson, a former student, utilized DRaCALA to detect interactions between proteins and larger nucleic acids, indicating that the assay may allow for the rapid detection of biomarkers and pathogens in the clinical setting. The development of this simple DRaCALA technique not only enabled me to answer the question I was personally interested in but has also reduced the technological barrier for scientists interested in other signaling molecules and binding proteins. As evidenced by my adventurous journey in science, support for basic research can pave the way for discovery and applications in unforeseeable ways. I believe continued support for basic research is critical to advance our knowledge and uncover innovative solutions to existing and new problems.

**Image 1:**
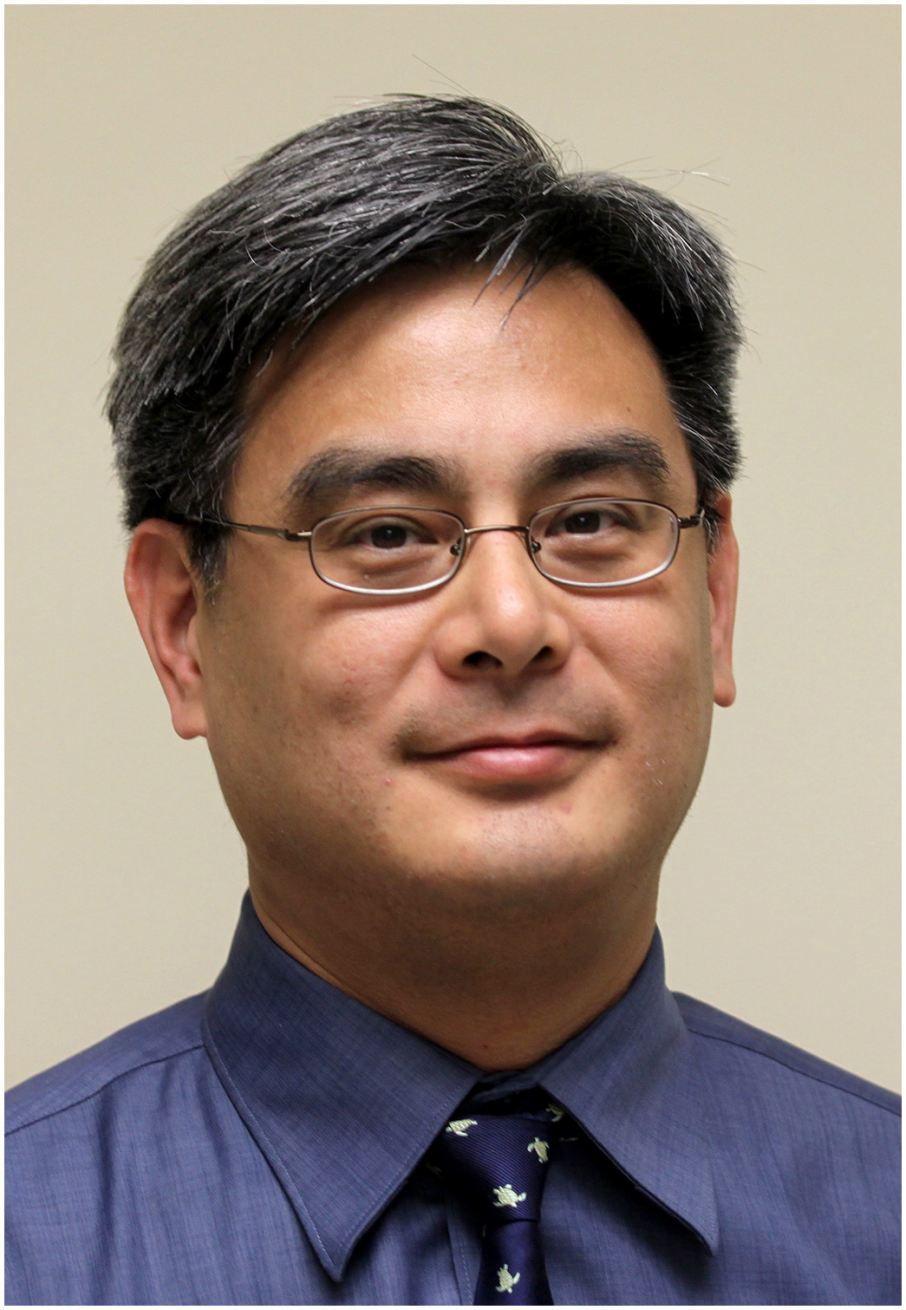
Vincent T. Lee.

